# Predictive capacity of FRAX in a spanish region with a hip fracture rate close to the national mean

**DOI:** 10.1186/s12891-023-06670-w

**Published:** 2023-07-15

**Authors:** Marta Zwart, Rafael Azagra-Ledesma, Marc Saez, Amada Aguyé-Batista, Miguel Angel Díaz-Herrera, Salvador Tranche-Iparraguirre

**Affiliations:** 1 Medicina de Familia. Centro de Atención Primaria Can Gibert del Pla, Institut Català de la Salut (ICS), C/ Sant Sebastià 50, Girona, 17006 Spain; 2 Departamento de Medicina, Universitat de Girona (UdG), C/ Emili Grahit 77, Campus Centro, Girona, 17003 Spain; 3 GROICAP. Unitat Suport a la Recerca (USR) Girona-IDIAP Jordi Gol, Girona, 17003 Spain; 4 Medicina de Familia. Centro de Atención Primaria Badía del Vallés, Institut Català de la Salut (ICS). C/ Bètica s/n, Badia del Vallès, Barcelona, 08214 Spain; 5 Departamento de Medicina, Universitat Autònoma de Barcelona, Avda Can Domènech, Bellaterra, Barcelona, 08193 Spain; 6 Fundación PRECIOSA para la Investigación, 08210 Barberà del Valles, Barcelona, Spain; 7 Bioestadística. Universitat de Girona (UdG), C/de la Universitat de Girona 10, Campus de Montilivi, Girona, 17003 Spain; 8 Grup de Recerca en Estadística, Econometria i Salut (GRECS), UdG y CIBER de Epidemiologia y Salud Pública (CIBERESP), Girona, 17003 Spain; 9 Medicina de Familia. Centro de Atención Primaria Granollers Vallés Oriental, Institut Català de la Salut (ICS). C/ Museu 19, Granollers, Barcelona, 08401 Spain; 10 Departamento de Medicina. Universitat Autònoma de Barcelona. Avda de Can Domènech, Bellaterra, Barcelona, 08193 Spain; 11 Enfermería. Unidad de Heridas Complejas Atención Primaria Metropolitana Sur. Institut Català de la Salut, Av. Mare de Déu de Bellvitge 3., Hospitalet de Llobregat. Barcelona, 08907 Spain; 12 Medicina de Familia. Centro de Salud El Cristo, Servicio Asturiano de Salud. C/ Álvaro Flórez Estrada 21, Oviedo, Asturias, 33006 Spain; 13 Comisión de Docencia. Hospital Universitario General de Catalunya-Grupo Quironsalud, C/ Pedro Pons 1, Sant Cugat del Vallès-Barcelona, 08195 Spain; 14 President of Sociedad Española de Medicina Familiar y Comunitaria (SemFYC), Barcelona, Spain

**Keywords:** FRAX®, Primary care, Osteoporotic fracture risk, Osteoporotic fracture

## Abstract

**Background:**

It is known that standardized incidence rates of hip fracture vary among older people in Spain. So far, the results published on the validation of the FRAX® tool in Spain have suggested that the major osteoporotic fractures (MOFs) risk in our country is underestimated. These studies have practically been based on Spanish cohorts evaluated in Catalonia, a higher hip fracture rate area. The purpose of this study is to analyse the ability of the FRAX® in a Spanish mid-fracture rate population.

**Methods:**

Study design: Retrospective cohort study.

**Measures:**

MOFs: hip, humerus, wrist, spine fractures. Risk of fracture assessed by calculating odds ratios (ORs). Predictive capacity of FRAX® according to the osteoporotic fractures observed between 2009 and 2018 (ObsFr) to predicted by FRAX® without densitometry in 2009 (PredFr) ratio.

**Results:**

285 participants (156 women, 54.7%) with a mean ± SD of 61.5 ± 14 years. Twenty-four people sustained 27 fractures (15 MOFs). Significant ORs were observed for an age ≥ 65 (2.92; 95% CI, 1.07–7.96), female sex (3.18; 95% CI, 1.24–8.16), rheumatoid arthritis (0.62; 95% CI, 2.03–55.55), proton pump (2.71; 95% CI, 1.20–6.09) and serotonin reuptake (2.51; 95% CI, 1.02–6.16) inhibitors. The ObsFr/PredFr ratio in women were 1.12 (95% CI, 0.95–1.29) for MOFs and 0.47 (95% CI, 0-0.94) for hip fractures. Men had a ratio of 0.57 (95% CI, 0.01–1.14) for MOF, no hip fractures were observed. The ratios for the overall group were 1.29 (95% CI, 1.12–1.48) for MOFs and 0.70 (95% CI, 0.22–1.17) for hip fractures.

**Conclusions:**

FRAX® accurately predicted MOFs in women population with a hip fracture incidence rate close to the national mean compared to previous studies conducted in higher incidence regions in Spain.

## Background

Fragility fractures have a significant impact on patient health and quality of life in terms of direct health effects, associated sequelae, loss of autonomy, and increased dependency. They also pose a major burden on health care systems [1].

Older age is one of the main risk factors for osteoporotic fractures, and is also linked to an increased prevalence of other risk factors, such as falls, a loss of bone mass and diseases that affect physical function [2, 3]. International experts and clinical practice guidelines recommend opportunistic detection of known risk factors to enable interventions that reduce the risk of osteoporotic fractures [3]. Some scientific associations also recommend assessing bone mineral density (BMD) by dual-energy x-ray absorptiometry (DXA) in patients of a certain age, particularly postmenopausal women [4]. Several tools for predicting osteoporotic fractures have been developed [5]. The FRAX® tool is the most widely used risk calculator internationally and its algorithms model the risk of osteoporotic fracture, expressed as the probability of sustaining a fracture over the next 10 years. The tool distinguishes between the risk of a major osteoporotic fracture (MOF), defined as a composite of hip (proximal femur), proximal humerus, distal forearm, and clinical spine fractures, and the risk of a hip fracture. The developers of the tool argue that this distinction is important because hip fractures are the most severe type of fracture and because many countries have epidemiological data on this but not other fractures [6]. Since the FRAX® tool was first released in 2008, numerous countries have undertaken new and updated studies to improve the predictive accuracy of the model and validate its performance using more recent country-specific fracture data.^4,7−11^ The earliest studies of the predictive capacity of FRAX® in Spain were conducted using data from three general population cohorts of women and the early results tended to underestimate MOF risk and provide acceptable estimates of hip fracture [7–9].

Two key aspects differentiate Spain from other countries in terms of early analyses of FRAX’s ability to identify individuals at high risk for osteoporotic fractures. First, the data used to test and analyse the predictive ability of the model were based on studies of women from just a few of the country’s regions [7] s, a number of epidemiological studies have reported significant differences between standardized incidence rates of hip fracture in different regions of Spain, with differences of over 50% in the most extreme cases [10]. Catalonia has one of the highest hip fracture rates in Spain and is where most FRAX® studies have been conducted [7, 9–11]. The rate in Asturias is close to the mean for the general Spanish population [10]. Asturias was one of the regions used to generate the algorithms for FRAX®-Spain, but no further studies have been performed there.

Considering the notable differences in the hip fracture incidence rates reported in different areas of Spain and the hypothesis that a population with a standardized incidence closer to the mean will provide a better scenario for assessing the predictive ability of the FRAX® tool, the main aim of this study was to analyse the tool’s ability to identify people at risk of osteoporotic fractures and examine associated risk factors. We focused particularly on women to enable comparisons with previous findings for Spain.

## Methods

### Study design

Retrospective cohort study.

### Setting

Primary care.

### Participants

General population from Asturias aged between 40 and 90 years in 2009 and enrolled in 2019.

### Sample size calculation

The EPIFROS-SPAIN project on risk factors for osteoporotic fractures was designed to include a sample of more than 4000 people from different regions of Spain that was representative of the general population aged ≥ 40 years (> 23 million people). According to census data from the National Statistics Institute, 632,308 people aged ≥ 40 years (2.7% of the Spanish population) were living in Asturias in 2009. In total, 110 people from Asturias were included in the sample size calculation for the country-wide project. Assuming a loss to follow-up of 30%, a power of 90%, and an alpha of 0.05, it was calculated that at least 143 people would be needed. Given the representative nature of all the regions and the number of general practitioners (GPs) who agreed to participate in the study, it was agreed to increase the sample size while maintaining the stratification by age and sex to reflect the demographics of the general population.

### Inclusion criteria

The study included Caucasian people living in Asturias who were aged between 40 and 90 years in 2009, selected from the patient lists of participating GPs in 2019.

### Exclusion criteria

Excluded were people who refused to participate, those who had died or moved to another region or had limitations that would have prevented their participation and/or whose relatives did not agree to answer the questionnaire. Also excluded were those who had received bone active drugs at baseline or during the 10-year follow-up period or had a missing or incorrect telephone number and those who did not answer three telephone calls made at different times.

### Variables

Baseline clinical variables and potential risk factors for osteoporotic fracture (included drug-induced osteoporosis) were collected in 2019 using a structured questionnaire. Information was collected on age, sex, previous fracture, parental history of hip fracture, active smoking, corticosteroid use, rheumatoid arthritis, intake of ≥ 3 standard units of alcohol/day, secondary osteoporosis according to the FRAX® criteria [6] in 2009, and information on fractures sustained during the previous 10 years (2009–2018, inclusive). MOFs and hip fractures were analysed separately, and information gathered from electronic records and a structured questionnaire administered to all patients. Only fractures consistent with osteoporotic fractures in the patients’ records and self-reported fractures supported by imaging tests or clinical reports were considered. Regarding drug induced osteoporosis (types and duration of medications taken) in 2009 and during follow-up period included corticosteroids, anticonvulsants, aromatase inhibitors, androgen deprivation, proton pump inhibitors (PPIs), selective serotonin reuptake inhibitors (SSRIs), pioglitazones, immunosuppressive drugs, antiretrovirals and loop diuretics.

The predictive ability of FRAX® was calculated as the ratio between the sum of observed fractures (ObsFR) during the period 2009–2018 and the sum of fractures predicted for this period by FRAX® (PredFR) in 2009. The latter was calculated as the sum of individual FRAX® risk scores without DXA [ObsFR/PredFR] for both MOFs and hip fractures.

### Data collection

The sample was randomly selected by the Health Area Technician and stratified using lists of patients assigned to each GP who agreed to participate in the study. The process included an appropriate representation from each age group based on their proportion in the population. Selected patients who visited their primary care center during the study period were directly invited to participate. Those without visits during this period were contacted by their GP. Once the individuals were selected, informed consent was requested, and individuals had to provide consent before being included in the study. The study was conducted in accordance with the work procedures and ethical standards of the research protocol, previously approved by the research committee at IDIAP Jordi Gol (P11/22). Data collection was carried for a duration of 6 months. The baseline variables in 2009 were collected from May to December 2019. Also, the information on drug-induced osteoporosis and fractures sustained prior 2009 and during the 10-year follow-up period (2009–2018), incident fractures were recorded and cross-validated using hospital and electronic records. To ensure reliable data, only fractures that were documented in both medical records and patients’ reports were considered.

### Statistical analysis

A descriptive analysis of the data was performed using frequencies and percentages for categorical variables and means, standard deviations (SD), and ranges for normally distributed quantitative variables. The distribution of risk factors for osteoporotic fractures was analysed and the strength of association between each factor and fracture risk was assessed using odds ratios (ORs) and 95% confidence intervals (95% CIs). Intervals that did not cross 1 were deemed equivalent to a significance of *P* < .05. The statistical analysis was performed in R, version 4.1.2.

## Results

Of the 345 randomly selected individuals, 19 (5.5%) refused to participate, 36 (10.4%) could not be contacted, and 5 (1.5%) had died. This left 285 individuals (82.6%) with a mean ± SD age of 61.5 ± 14 years; 156 (54.7%) were women.

The distribution of risk factors for osteoporotic fractures of any type is shown in Table 1. The ORs analysis of risk factors included in the FRAX® tool were significant for an age ≥ 65 years, female sex, and rheumatoid arthritis. In the analysis of risk factors not included in FRAX®, including long-term drug induced osteoporosis use (≥ 3 months), the ORs were significant only for PPIs and SSRIs.

**Table 1:** Risk factors for osteoporotic fractures of any type (2009–2018)

	Fracture	No fracture	OR	95% CI
Age ≥ 65 y	22	155	2.92	1.07–7.96
Female sex	21	135	3.18	1.24–8.16
Body mass index (kg/m^2^) < 20	1	9	1.06	0.13–8.73
Personal history of fracture	3	10	3.10	0.79–12.03
Parental history of hip fracture	1	22	0.41	0.05–3.18
Current smoking	4	81	0.38	0.12–1.13
Corticosteroids	1	8	1.37	0.16–11.65
Rheumatoid arthritis	3	3	10.62	2.03–55.55
Alcohol ≥ 3 units/day	2	17	1.13	0.24–5.19
Secondary osteoporosis^*^	1	6	1.61	0.18–13.94
**Morbidity**				
Ankylosing spondylitis	1	3	5.52	0.54–56.10
Type 2 diabetes mellitus	3	21	2.52	0.67–9.47
Chronic kidney failure	2	20	1.65	0.35–7.74
Body mass index (kg/m^2^) > 30	6	46	1.31	0.50–3.44
**Drugs used ≥ 3 months**				
Proton pump inhibitors	16	90	2.71	1.20–6.09
Selective serotonin reuptake inhibitors	8	37	2.51	1.02–6.16
Pioglitazone	0	0	NA	NA
Anti-seizure drugs	0	2	NA	NA
Antiretrovirals	0	1	NA	NA
Immunosuppressants	0	0	NA	NA
Loop diuretics	0	15	NA	NA
Aromatase inhibitors	0	2	NA	NA
Gonadotropin-releasing hormone inhibitors	0	6	NA	NA

Abbreviations: 95% CI, 95% confidence interval; NA, not available; OR, odds ratio

*Secondary osteoporosis according to the FRAX® tool includes type 1 (insulin-dependent) diabetes mellitus, adult osteogenesis imperfecta, untreated long-standing hyperthyroidism, hypogonadism, premature menopause (< 45 years), malnutrition or malabsorption, and chronic liver disease

Twenty-four patients sustained 27 osteoporotic fractures during the period 2009–2018 (2 patients sustained 3 subsequent fractures) (Table 2). Fifteen of the initial fractures (62.5%) were MOFs according to the FRAX criteria and 13 of them (86.7%) occurred in women. The other nine (37.5%) were fractures at sites not considered by FRAX®, and six of them (66.7%) were in women. Thirteen (86.7%) of the MOFs occurred in individuals aged ≥ 65 years (11 women, 84.6%). The three cases of subsequent fractures (12.5%) involved a woman aged ≥ 65 years who sustained a spine fracture and a hip fracture 4 and 8 years after fractures in similar locations and a man aged ≥ 65 years who sustained a rib fracture 3 years after a spine fracture.

**Table 2:** Fractures observed in the EPIFROS-Asturias cohort from 2009 to 2018

	Hip	Clinical spine	Distal forearm	Proximal humerus	MOF	Sites not included in FRAX	Subsequent fractures*	TOTAL
**Women**								
Age < 65 y	0	0	1	1	2	2	0	4
Age ≥ 65 y	2	2	6	1	11	4	2	17
Total	2	2	7	2	13	6	2	21
**Men**								
Age < 65 y	0	0	0	0	0	1	0	1
Age ≥ 65 y	0	1	0	1	2	2	1	5
Total	0	1	0	1	2	3	1	6
**Women and men**								
Age < 65 y	0	0	1	1	2	3	0	5
Age ≥ 65 y	2	3	6	2	13	6	3	22
Total	2	3	7	3	15	9	3	27

Abbreviations: MOF, major osteoporotic fracture (hip, clinical spine, proximal humerus, distal forearm)

* Fractures sustained in 2009–2018 following an initial fracture in the same period

The mean ten-year probability for major osteoporotic fracture was 4.1% and, for a hip fracture was 1.5%.

The predictive ability of FRAX® without DXA for MOFs and hip fractures stratified by sex and age is shown in Table 3. The ObsFR:PredFR ratio for MOFs was 1.12 (95% CI, 0.95–1.19) for women and 0.57 (95% CI, 0.01–1.14) for men. The corresponding ratios for women aged < 65 years and ≥ 65 years were 2.19 (95% CI, 0.01–4.39) and 1.53 (95% CI, 1.25–1.80), respectively. The ratios for hip fractures were 0.47 (95% CI, 0-0.94) for women of all ages and 0.71 (95% CI, 0-1.43) for women aged ≥ 65 years. None of the men sustained a hip fracture.

**Table 3:** Ability of the FRAX® tool to predict osteoporotic fractures without densitometry in the EPIFROS-Asturias cohort

	ObsFr	PredFr	ObsFr/PredFr	95% CI
	Major osteoporotic fractures*			
**Women (n = 156)**	13	11.60	1.12	0.95–1.29
< 65 y (n = 57)	2	0.91	2.19	0.01–4.39
≥ 65 y (n = 99)	11	7.21	1.53	1.25–1.80
**Men (n = 129)**	2	3.52	0.57	0.01–1.14
< 65 y (n = 51)	0	0.71	NA	NA
≥ 65 y (n = 78)	2	2.81	0.71	0.01–1.43
**Women and men (n = 285)**	15	11.6	1.29	1.12–1.48
< 65 y (n = 108)	2	1.62	1.23	0.01–2.47
≥ 65 y (n = 177)	13	10.01	1.30	1.10–1.50
	**Hip fractures**			
**Women (n = 156)**	2	4.24	0.471	0-0.94
< 65 y (n = 57)	0	0.09	NA	NA
≥ 65 y (n = 99)	2	2.79	0.714	0-1.43
**Men (n = 129)**	0	1.35	NA	NA
< 65 y (n = 51)	0	0.05	NA	NA
≥ 65 y (n = 78)	0	1.29	NA	NA
**Women and men (n = 285)**	2	4.24	0.47	0-0.94
< 65 y (n = 108)	0	0.14	NA	NA
≥ 65 y (n = 177)	2	4.09	0.48	0-0.97

Abbreviations: 95% CI, 95% confidence interval; NA, not available; ObsFr, observed fractures (2009–2018); PredFr, fractures predicted by the FRAX® tool in 2009; ObsFr/PredFr, ratio between observed (2009–2018) and predicted (2009)

*Composite of hip, clinical spine, proximal humerus, and distal forearm fractures

## Discussion

Not all the risk factors included in the FRAX® tool were significant predictors of osteoporotic fracture in the EPIFROS-Asturias cohort. Significant factors were an age ≥ 65 years, female sex (independent of age), and rheumatoid arthritis. Our findings are consistent with most previous reports [1, 11]. Contrasting with the findings of FROCAT-Catalonia, [12] an age < 65 years was not predictive in our cohort, probably because of the considerably smaller sample size. Although long-term use of certain drugs has been described as a risk factor for osteoporotic fracture in the literature, it is not assessed in the FRAX® tool. PPIs and SSRIs, which are widely used in the older general population, were significant predictors of fracture risk in the EPIFROS-Asturias cohort, positioning them as potential candidates for inclusion in future versions of predictive models, to give an example, as the EPIC risk algorithm and others have done with the antidepressants [13, 14]. Type 2 diabetes has been linked to an increased risk of osteoporotic fracture, included in early stages of the disease [15]. This factor was not significant in our cohort. The lack of significance observed for it and other risk factors with significant associations in previous studies in Spain [7, 16, 12] could be related to a lower incidence of fractures in our cohort or its smaller size.

Of the osteoporotic fractures observed between 2009 and 2018 in the cohort, 75% occurred in women and 80% in men and women aged ≥ 65 years. These results are consistent with most findings to date [7]. The FRAX® tool assesses just four fracture sites, yet more than a third of the fractures in our cohort and others [1, 11] occurred elsewhere. The decision by the FRAX® creators to focus on just four sites was based on several factors, including the frequency and severity of fractures at these sites and the well-established association with loss of BMD [6].

There has been much discussion about when subsequent osteoporotic fractures are most likely to occur, with some recent studies suggesting that imminent subsequent fracture risk is highest in the 2 years after an initial fracture [17, 18]. In the EPIFROS-Asturias cohort, we observed an overall subsequent fracture rate of 12.5% after 3 years.

The ObsFR/PredFR ratio calculated to estimate the ability of the FRAX® tool to predict osteoporotic fractures without DXA was close to 1 for MOFs in women, with no significant differences observed overall. This contrasts with findings from early studies that, using the same ratio, found that women sustained almost twice as many osteoporotic fractures as those predicted by the tool [7–9, 16, 12]. These early findings led several experts to question the use of FRAX® in the Spanish population [8, 9]. Studies supporting the creation of algorithms to predict osteoporotic fracture were published almost 10 years before the release of the FRAX® tool, in 2008 [6]. In addition, significant changes in hip fracture incidence trends have been observed in several countries, including Spain [19, 20]. We chose Asturias as our study region, not only for convenience and feasibility reasons, but also because its standardized hip fracture incidence rate is close to the Spanish mean. The two relevant cohort studies that first applied the FRAX® tool in Spain, by contrast, have analysed the tool in the Catalan population, which has a hip fracture rate on the high side of the mean [9, 10]. It is therefore possible that findings generated by studies to date, limitations notwithstanding, have generated conflicting views on the ability of the Spanish model to accurately predict osteoporotic fracture risk and guide decisions on interventions [8, 9]. Logically, such stances have fuelled debate and differing opinions that are reflected in publications and clinical practice guidelines [1, 4].

In brief, there are arguments for and against the use of the FRAX® tool in Spain. Detractors point to early findings showing a tendency towards underestimation of osteoporotic fracture risk and also draw attention to the limitations of these studies [8, 9] and subsequent proposals to improve the FRAX® algorithms [16, 12] They also mention the limited number of risk factors assessed in the tool and stress that algorithms are no substitute for expert clinical judgement. Supporters of the tool, by contrast, while aware of the limitations of the early studies and proposed calibrations, [4, 16, 12] claim that the greatest benefit of FRAX® is its contribution to reducing variability in clinical practice by guiding decision-making by non-experts, especially in primary care. They also believe that it enables a more rational use of pharmacologic and non-pharmacologic interventions for primary and secondary fracture prevention.

Contrasting with previous findings on the ability of the FRAX® tool to predict osteoporotic fractures among women in Spain (primarily Catalonia), we found no significant differences between real and predicted fractures among women in the EPIFROS-Asturias cohort. This is important, because both the CETIR [9] and FROCAT [12] cohorts, which used a similar methodology, detected significant differences. Possible reasons for the conflicting results are geographic effects and differences in mortality, as more deaths mean fewer fractures. Other likely reason is that the data used to create FRAX®-Spain came from the European Vertebral Osteoporosis Study, which analysed populations in four Spanish regions: Asturias, Catalonia, Madrid, and the Canary Islands. Although Asturias was the most heavily represented region, the overall response to questionnaires was low [20]. To date, the only finding that has been demonstrated and confirmed is that standardized incidence rates of hip fracture vary among older people across Spain [10, 19]. It would therefore seem logical to assume that the differences observed between the predictive capacity of FRAX® in the EPIFROS-Asturias cohort study and earlier studies are related to differences in hip fracture rates.

This study has strengths and limitations. Among its strengths are a follow-up time of 10 years, randomization, and the dual collection methodology (interviews and chart reviews) used to reduce the risk of erroneous information due to recall lapse and underreporting. Another strength is our choice of region, Asturias, which has a standardized osteoporotic fracture incidence rate close to the Spanish mean and was included in the studies used to create the algorithms for the FRAX®-Spain tool. Limitations of this study include its small sample size and retrospective design and the use of data from medical records designed for routine clinical practice, not research. Recall bias is particularly common in epidemiological studies involving older people, although this risk was minimized by the dual collection method. From an epidemiological perspective, it is worth noting that mortality competes with osteoporotic fracture risk over time in the FRAX® tool [21]. We did not adjust for mortality, as although we detected deaths among the population selected to participate, we were unable to determine the vital status of those who could not be contacted. To our knowledge, none of the epidemiological studies that have analysed hip fracture incidence in Spain have adjusted for mortality [10, 19]. Finally, validation studies of the FRAX® tool have provided an opportunity to study the epidemiology of osteoporotic fractures in numerous countries, [22] including Spain. A greater understanding of the limitations of the tool has prompted experts in fracture risk to stress the need for reasoned clinical judgment and consideration of additional risk factors, including long-term use of drugs such as aromatase inhibitors, PPIs, and SSRIs. Fracture risk, however, must also sometimes be assessed by physicians who have less expertise and familiarity with risk factors than metabolic bone specialists, such as GPs, gerontologists, and gynecologists. FRAX® is a simple, readily accessible tool that can guide decision-making in such cases. This is perhaps the tool’s main advantage: its ability to help non-experts, make better, more informed decisions in daily practice. Also, it should not be forgotten that osteoporotic fracture risk calculations are closely linked to epidemiological data. Although the FRAX® tool will certainly need fine-tuning as new data emerge, it offers a more objective and less variable measure of risk in clinical practice as well as more accurate estimates than decisions based solely on age and sex or BMD measured by DXA [23].

## Conclusions

In conclusion, the predictive ability of the FRAX® tool assessed in women from the EPIFROS-Asturias cohort was slightly higher than 1, with significant differences observed only for women aged ≥ 65 years. In other words, the tool did not overestimate the 10-year risk of osteoporotic fractures in the general female population. Ours is the first study to show accurate predictions by FRAX® in women. In previous studies conducted in Spain, the FRAX® tool predicted less than half of all osteoporotic fractures sustained by women, but they were conducted in regions with a higher standardized incidence rate of hip fractures [8, 9, 12].

Our findings should be interpreted within the context of the relatively low number of fractures observed, especially hip fractures. Further research is needed to identify new factors to include in risk calculators and guide decisions on adjustments to existing factors. More studies are also needed to gather data on osteoporotic fractures in representative general populations in different regions of Spain.

## Data Availability

The datasets used and/or analysed during the current study are available from the corresponding author on reasonable request.

## Abbreviations

BMD: bone mineral density
DXA: dual-energy x-ray absorptiometry
GPs: general practitioners
MOF: major osteoporotic fracture
NA: not available
ObsFr: observed fractures
OR: odds ratio
PPIs: proton pump inhibitors
PredFr: predicted fractures
SD: standard deviation
SSRIs: selective serotonin reuptake inhibitors
95% CI: 95% confidence intervals
